# Popular Education in Dentistry

**Published:** 1896-05

**Authors:** 


### Popular Education in Dentistry.

A paper on this subject was read at the recent meeting of the Mississippi Valley Dental Society in which some valuable suggestions were presented. It deals mainly with the methods of accomplishing this work, and some very interesting—and in certain directions very practicable—thoughts are presented. This has, ever since the establishment of the profession, been a subject of interest with dentists. The subject has been discussed in nearly all dental societies. At the last meeting of the American Dental Association a committee was appointed to take the matter into consideration and devise and arrange for carrying out some scheme looking to this end. Various methods of accomplishing this object have been suggested in the past, such as giving information by popular lectures, introducing proper dental information into schools—either by lectures or text-books or both—and by the publication of popular treatises (these to be distrbuted by dentists generally), and by each and every dentist constituting himself a teacher in this line so far as he might be disposed and have the ability. Dr. Bethel, in the paper referred to, deals chiefly with the public press as an avenue through which this work would be practically carried out, and he gives in that article (which may be found on page 234 of the present issue), somewhat of the details of the method. If these could be carried out and could have the full co-operation of the profession generally, there is no doubt that great good would be accomplished. We think it true, however, that the various methods referred to may each and every one be valuable if wisely employed, and we would suggest that every dentist study this subject for himself, carefully reading Dr. Bethel's paper, and then work on this line energetically in whatever way seems to himself most feasible. Perhaps any of these methods, if adopted heartily by the profession, could be made to accomplish very great good, both for the public and the profession.

In the discussion of this subject the position was taken that the ignorant patient was the best and most desirable one. This position, however, did not find a hearty response from those who were present.

